# *De novo* Assembly, Annotation, and Analysis of Transcriptome Data of the Ladakh Ground Skink Provide Genetic Information on High-Altitude Adaptation

**DOI:** 10.3390/genes12091423

**Published:** 2021-09-16

**Authors:** Sylvia Hofmann, Chitra Bahadur Baniya, Matthias Stöck, Lars Podsiadlowski

**Affiliations:** 1 Centre of Taxonomy and Evolutionary Research, Zoological Research Museum Alexander Koenig, D-53113 Bonn, Germany; 2 Department of Conservation Biology, UFZ—Helmholtz-Centre for Environmental Research, D-04318 Leipzig, Germany; 3 Central Department of Botany, Tribhuvan University, Kirtipur 44618, Nepal; chitra.baniya@cdb.tu.edu.np; 4 Department of Ecophysiology and Aquaculture, Leibniz-Institute of Freshwater Ecology and Inland Fisheries, D-12587 Berlin, Germany; matthias.stoeck@igb-berlin.de; 5 Amphibian Research Center, Hiroshima University, Higashihiroshima 739-8526, Japan; 6 Centre for Molecular Biodiversity Research, Zoological Research Museum Alexander Koenig, D-53113 Bonn, Germany; l.podsiadlowski@leibniz-zfmk.de

**Keywords:** adaptation, evolution, genomic, high elevation, Himalayas, Scincidae

## Abstract

The Himalayan Arc is recognized as a global biodiversity hotspot. Among its numerous cryptic and undiscovered organisms, this composite high-mountain ecosystem harbors many taxa with adaptations to life in high elevations. However, evolutionary patterns and genomic features have been relatively rarely studied in Himalayan vertebrates. Here, we provide the first well-annotated transcriptome of a Greater Himalayan reptile species, the Ladakh Ground skink *Asymblepharus ladacensis* (Squamata: Scincidae). Based on tissues from the brain, an embryonic disc, and pooled organ material, using pair-end Illumina NextSeq 500 RNAseq, we assembled ~77,000 transcripts, which were annotated using seven functional databases. We tested ~1600 genes, known to be under positive selection in anurans and reptiles adapted to high elevations, and potentially detected positive selection for 114 of these genes in *Asymblepharus*. Even though the strength of these results is limited due to the single-animal approach, our transcriptome resource may be valuable data for further studies on squamate reptile evolution in the Himalayas as a hotspot of biodiversity.

## 1. Introduction

The Himalayan arc represents one of the world’s most important faunal and floral hotspots with high species diversity and endemism [1], which result from the Tertiary orogeny of this mountain chain, its complex topography as well as its great climatic heterogeneity and isolation. The genesis of the Tibetan highlands and the Himalayas since the Paleogene, with the Greater Himalayas starting to rise presumably the earliest in the post-Eocene (for a review, see the supplementary in Hofmann et al. [2]), triggered the evolution of unique biodiversity under gradual high-altitude adaptation, as already shown for anurans [3,4,5,6,7]. Besides amphibians, there are also several reptiles that can cope with life at high altitude in those regions, e.g., *Thermophis* [8], *Phrynocephalus* [9], and some *Laudakia* species [10]. Potential constraints to upslope migration of reptiles (and amphibians) to high-elevation environments are the substantial UV-radiation, the thermal extremes, and especially the oxidative stress, referred to as high-altitude hypoxia, which interacts with temperature in a context-dependent manner to influence thermal performance and limits in terrestrial ectotherms [11,12]. Recent advances in high-throughput sequencing technologies have led to a growing number of genomic studies that address the molecular basis of high-altitude adaptation, some of them focused also on reptiles [13,14,15]. However, such data have been scarce in non-model species of the Greater Himalayas (but see [16]). This results from the general understudied biodiversity of this high-mountain range, presuming a relatively large number of cryptic and undiscovered species [17], even among vertebrates. Molecular data from Himalayan organisms can contribute to understanding of the taxonomic and functional diversity spectra across this species-rich, fragile ecosystem. These data resources are even more important because Himalayan biodiversity is threatened at the very core; rapid warming due to climate change, especially at higher elevations, as well as higher rates of forest degradation and deforestation, infrastructural development, trade routes, and hydropower dams are driving species loss at a very alarming speed [18,19]. To allow future studies in evolutionary biology at a genomic level and to generally provide sufficient and relevant data for Himalayan reptiles, in the present study, we have generated a new genomics data set based on RNAseq for a scincid species from the Greater Himalayas. Using these data, we specifically aimed to identify genes known to play roles in adaptation of terrestrial ectothermic vertebrates to high elevations. Since exposure to oxidative stress can particularly affect the physiology during early development [20] and in oxygen-sensitive organs [21,22], such as the nerve system, we focused on embryonic and brain tissue samples.

**Figure 1:**
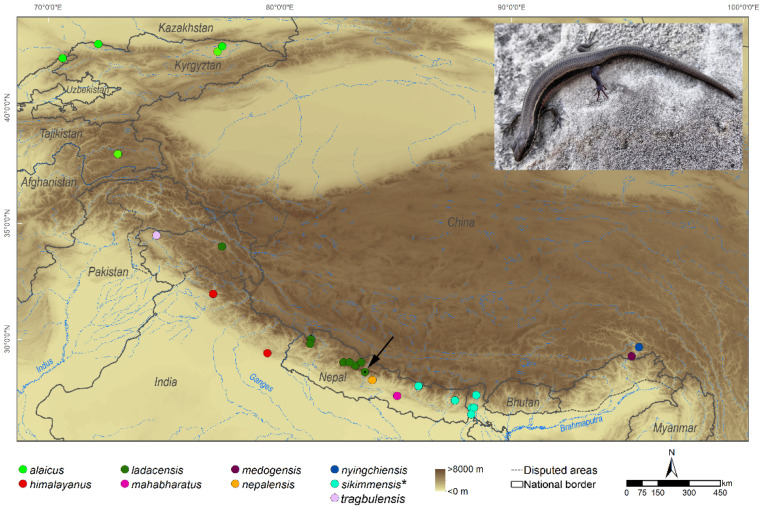
Map of *Asymblepharus* species based on GBIF (www.gbif.org; accessed on 20 July 2021) records of preserved specimens and georeferenced localities in the taxonomic reptile database (https://reptile-database.reptarium.cz/; accessed on 20 July 2021). The location of our RNA sample of the female *A. ladacensis* (photo) is indicated by a green circle with a dot in the middle and an arrow. * Note, according to a large-scale phylogeny of squamates, *A. sikimmensis* is nested within *Scincella*; however, it remains unclear whether this single “*A. sikimmensis*” specimen, on which the sequence data are based, had been taxonomically correctly identified. Therefore, we also show the GBIF records of specimens collected as *A. sikimmensis*. Records of *A. eremchenkoi* in the databases could not be georeferenced due to insufficient information on the collection site. Photo credit: Sylvia Hofmann.

Our target species is a scincid lizard in the genus *Asymblepharus*, the Ladakh Ground Skink, *A. ladacensis* (GÜNTHER, 1864), which is endemic to the western part of the Himalayas. The genus further contains the following species (Figure 1): *A. alaicus* (ELPATJEVSKY, 1901), *A. eremchenkoi* PANFILOV, 1999, *A. himalayanus* (GÜNTHER, 1864), *A. mahabharatus* EREMCHENKO, SHAH & PANFILOV, 1998, *A. nepalensis* EREMCHENKO & HELFENBERGER, 1998, and *A. tragbulensis* (ALCOCK, 1898). Another two species, *Asymblepharus medogensis* JIANG, WU, GUO, LI & CHE, 2020 and *A. nyingchiensis* JIANG, WU, WANG, DING & CHE, 2020, have been described very recently from Mêdog, Nyingchi in SE Tibet, China. According to a large-scale phylogenetic study of squamates [23], the sampled specimen of *A. sikimmensis* (BLYTH, 1854) is nested within *Scincella* and was therefore suggested to be transferred to this genus. However, it remains unclear whether this single specimen had been misidentified as *A. sikimmensis* since originally it was labeled in the museum collection as *Scincella potanini* (voucher catalogue number CAS:HERP:194923, see http://portal.vertnet.org/o/cas/herp?id=urn-catalog-cas-herp-194923 (accessed on 29 July 2021) [24].

In general, *Asymblepharus* is a genus with a still poorly known endemic distribution, origin, and evolutionary history. No studies of its population genetic structure and genetic diversity exist to date for any *Asymblepharus* taxon, and the current taxonomic relationships of its lineages are in flux [23,24,25]. The three Himalayan species *A. himalayanus*, *A. ladacensis*, and *A. tragbulensis* have frequently been assigned to the genus *Himalblepharus* Eremchenko, 1987. According to literature data ([26] and references therein) and personal observation, they show a remarkably wide vertical distribution from the foothills (~150 m a.s.l.) to the high alpine zone and even up to the snow line (~5500 m a.s.l.), making them an excellent model to study the genetic basis of adaptations to high altitudes in ectotherms and the evolutionary processes accounting for them.

With this paper, we characterize the first transcriptome data set of a high-altitude reptile species from the Greater Himalayas and report genes known to play roles in adaptation of ectotherms to high elevations of this non-model reptile. Such data provide the necessary sources for future molecular studies in Himalayan reptiles and high-altitude vertebrates.

## 2. Materials and Methods

### 2.1. Sample Collection and Ethics Statement

A single gravid female *Asymblepharus ladacensis* was collected in Central Nepal, in the Dhaulagiri range, west of the Kali Gandaki valley (28.68° N, 83.59° E; 2714 m a.s.l.; Figure 1). Samples were collected in accordance with regulations for the protection of terrestrial wild animals under the permits of the Nepal expeditions of the Natural History Museum of Erfurt, Germany [27,28]. All treatments were carried out in accordance with approved guidelines and according to the permit as well as the local animal welfare committee’s instructions (VNME 17, 15–30). Tissues were transferred into RNAlater (Thermo Fisher), kept at ambient temperature during the time of the fieldwork, and later stored at −30 °C.

### 2.2. RNA Isolation, Library Preparation, and Sequencing

We followed the same procedure as previously described [16]. In brief, total RNA was isolated from the brain, an embryonic disc, and from pooled tissues (including lung, muscle, and heart) using TRIzol Reagent (Thermo Fisher Scientific, Waltham, MA, USA) according to the supplier’s recommendation, in combination with the RNeasy Mini Kit (Qiagen, Hilden, Germany) and adjusted to equal concentrations. RNA quality was assessed by RNA concentration, RIN (RNA Integrity Number) value, 28S/18S and fragment length distribution using an Agilent Bioanalyzer (Agilent Technologies, Inc., Santa Clara, CA, USA).

Complementary DNA (cDNA) library preparation and paired-end sequencing were carried out by BGI (BGI-Hongkong Co., Ltd., Tai Po District, Hong Kong), using Illumina NextSeq500 sequencing system (Illumina, San Diego, CA, USA). The raw reads quality was examined using FastQC v0.11.9 [29].

### 2.3. Assembly and Assessment of Transcriptome Quality and Completeness

First, we controlled our data for rRNA quantity using SortMeRNA 4 [30]. The three *de novo* assemblies were then created following the Oyster River Protocol (ORP; Docker image 2.2.8) best practices [31]. This protocol implements both pre-assembly procedures and a number of different kmer lengths and assemblers, finally merging these assemblies into a single, comprehensive transcriptome. The rationale behind it is that assembling RNAseq reads with different assembly tools increases assembly quality and mapping rate and, in turn, the ability to draw conclusions from that fraction of the sample [31]. Thus, merging the contigs resulting from several assemblers and parameter configurations to combine the advantages of certain assembly mechanisms and to overcome their different disadvantages seems to be the best way to obtain a comprehensive *de novo* transcriptome assembly [32,33].

Illumina sequencing adapters and nucleotides with quality Phred ≤ 2 were removed using Trimmomatic v0.36 [34], then the reads were error corrected by Rcorrector version 1.0.2 [35]. These reads were then assembled using Trinity release 2.8.4 [36] with default settings (*k* = 25), two independent runs of SPAdes assembler version 3.11 with kmer lengths of 55 and 75 [37], and the assembler Shannon version 0.0.2 with a kmer length of 75 [38]. The resulting four distinct transcriptome assemblies were then merged to a single, comprehensive transcriptome using Ortho-Fuser [31]. This final transcriptome was evaluated with TransRate version 1.0.3 [39], which is modified for and packaged with the ORP, and with BUSCO (Benchmarking Universal Single-Copy Orthologs) version 4.1.4 [40,41]. Searching the assembly for conserved single-copy orthologs found in orthologous sets of genes constructed from genomes representing eukaryotes (70 species: 255 BUSCOs), vertebrates (67 species: 3354 BUSCOs), and tetrapods (38 species: 5310 BUSCOs).

A detailed quality assessment of the assembly with respect to known genes was further obtained with rnaQUAST version 2.2.0 [42] using the reference genomes of *Anolis carolinensis*, *Gekko japonicus*, and *Python bivittatus*.

### 2.4. Functional Annotation

Transcripts were functionally annotated as previously described [16]. Briefly, sequence homology searches were conducted against seven databases (Gene Onthology, GO; Kyoto Encyclopedia of Genes and Genomes, KEGG; EuKaryotic Orthologous Groups, KOG; InterPro; the non-redundant nucleotide database, NT; the non-redundant protein database, NR; SwissProt). To align our data to KEGG, KOG, NR, and NT and SwissProt, we used Diamond v0.8.31 [43] or the BLASTx [44] algorithm; matched transcripts were filtered by using a cut-off *e*-value of 1 × 10^−25^. Transcripts that aligned to the NR database were transferred to the GO database with Blast2GO v2.5.0 [45] and assigned into the following three groups: biological process, cellular components, and molecular functions. InterProScan5 v5.11-51.0 tool [46] was used to annotate against the InterPro databases. Blast v2.2.23 was used to search in SwissProt and hmmscan v3.0 [47] for search against Pfam database (for each sample individually). Candidate coding areas within the transcript sequences were predicted by TransDecoder v.3.0.1 [36]. For a coding sequence with multiple open reading frames (ORF), the longest one was selected. We also used the getorf program of the EMBOSS v6.5.70 package [48] to find the ORF of each transcript and mapped them to the Animal Transcription Factor DataBase (AnimalTFDB2.0). The threshold of transcript lengths used for annotation and downstream analyses was ≥200 bp.

### 2.5. Positively Selected Genes Related to Mechanisms of High-Altitude Adaptation

We selected transcripts of genes reported to exhibit molecular adaptation to high elevations in lizards and anurans as provided in the literature [7,15]. This comprised a total of 143 genes identified to be under positive selection (PSG) in high-elevational lineages of lizards (*Phrynocephalus vlangalii*) [15]. We included further 1481 PSGs of these lizards (*P. erythrurus, P. putjatia*, *P. vlangalii*) and of dicroglossid frogs (*Nanorana*, *Quasipaa*) [7], genes that were identified across an elevational gradient (~1000 m to 4500 m). These additional, individual genes were grouped according to the phylogenetic tree branches across the elevational gradient as presented in Sun et al. (2018) [7]: PSGs attributed to branches that represent (i) lowland species (~1000 m), (ii) species distributed in colline zones up to about 2000 m, (iii) submontane and montane species (2000 and 3500 m), and (iv) subalpine and alpine species (>3500 and 4500 m). Given the vertical distribution of *Asymblepharus ladacensis* in the Himalayas between ~2500 m and 4500 m [49], we expected to find primarily genes under positive selection reported as PSGs for the submontane and montane, as well as the subalpine and alpine species.

To identify scincid orthologs to the genes under positive selection (high-altitude adaptations) in reptiles and amphibians, we used the corresponding coding sequences from *Anolis carolinensis* (AnoCar2.0, gene build from ensemble 104.2; we used *Anolis* gene numbers that were mentioned in the publications cited in the last paragraph) as a reference for blast searches. We performed blast searches with the newly sequenced and assembled transcriptome of *A. ladacensis*, as well as with publicly available transcriptome data from the following scincid lizards: *Scincella lateralis* (NCBI-SRA SRR629642), *Lampropholis guichenoti* (NCBI-SRA SRR4293354), *Lepidothyris fernandi* (NCBI-SRA SRR10360868), and *Pseudemoia entrecasteauxii* (NCBI-SRA SRR3099521). Only the best reciprocal hit between *Anolis* and each scincid species (and only if the *e*-value was below 10^−25^) were used for subsequent analyses.

Alignments of orthologs to the *Anolis* reference were done with mafft v.7.455 [50] making use of the “adjustdirection” and “keeplength” options to get alignments that are in the same direction and keep reading frames intact. Alignments were inspected for good representation of all species under study. When one or more species had substantially shorter, incomplete contigs as best reciprocal hits, we omitted those from the alignment. A phylogenetic tree for each alignment was produced using FastTree v. 2.1.10 [51] with default settings, except for the nucleotide option.

Alignments and trees were analyzed with the HyPhy (Hypothesis Testing using Phylogenies) package v. 2.5 [52,53] using the following methods (for details see [54]): (i) The BUSTED (Branch-Site Unrestricted Statistical Test for Episodic Diversification) [55] model, to test whether a given gene has been subject to positive, diversifying selection at any site, at any time (we tested all lineages for positive selection); (ii) FUBAR (Fast, Unconstrained Bayesian AppRoximation) [56], a Bayesian approach to infer which site(s) in a gene are subject to pervasive, i.e., consistently across the entire phylogeny, diversifying selection (we considered a posterior probability of at least 0.90 as significant); and (iii) the branch-site model aBSREL (adaptive Branch-Site Random Effects Likelihood) [57,58], to test whether codon sites and individual branches are subject to positive selection across the phylogeny. The threshold for significance in BUSTED and aBSREL was set at a *p*-value lower than 0.05.

GO term and metabolic pathway enrichment analysis was done using pantherdb (pantherdb.org; accessed on 2 August 2021, [59]), using the genome of *Anolis carolinensis* as reference.

### 2.6. Data Availability

Data generated in this study are publicly available from the NCBI GenBank database under the Bioproject ID PRJNA750278, BioSamples SAMN20458631, SAMN20458632, and SAMN20458631. All sequence data were deposited in the NCBI Sequence Read Archive (SRA, http://www.ncbi.nlm.nih.gov/Traces/sra/; accessed on 2 August 2021) under the accession numbers SRR15283177, SRR15283178, and SRR15283179’; assembled sequences were transmitted to NCBI Transcriptome Shotgun Assembly Sequence Database (TSA, http://www.ncbi.nlm.nih.gov/genbank/tsa; accessed on 2 August 2021).

## 3. Results

### 3.1. Sequencing and Transcriptome Assembly

All RNA was reasonably high quality; A260/280 ratios ranged between 1.81 (brain), 1.76 (embryonic disc), and 2.09 (pooled tissues); RIN values were between 7.90, 8.20, and 9.10, respectively. RNA-seq libraries of the two tissues yielded a total of 74.78, 73.56, and 64.20 million raw sequence reads (Table 1). Pre-processing of reads via read trimming and read error correction removed approximately 2–4% of the raw data, resulting in 73.14, 71.89, and 61.40 million clean reads for the brain, embryonic disc, and pooled tissues (Table 1). GC content of these clean reads was 48%.

The final *de novo* assemblies generated from ORP resulted in 151,718 (brain), 105,133 (embryonic disc), and 66,696 (pooled tissues) transcripts with a total length of ~102.61, ~98.92, and ~47.61 million bp, respectively. Transcripts had an average length of 676 bp (brain), 940 bp (embryonic disc), and 712 bp (pooled tissues), and an N50 of 1215 bp, 2052 bp, and 1194 bp (Table 1, Supplementary Table S1 and Figure S1). A total of 48,884 (32.22%; brain), 44,358 (42.19%; embryonic disc), and 25,772 (38.64%; pooled tissues) transcripts were longer than 500 bp (Supplementary Table S2).

### 3.2. Assembly Completeness

TransRate’s optimal assembly score (min 0.0, max 1.0) is considered to be a good parameter of the quality of an assembly [32]; it captures the confidence and completeness of the assembly. The TransRate scores of our final assemblies were high, ranging between 0.44 (optimized score 0.49) for the brain sample, 0.45 (optimized score 0.57) for the embryonic disc sample, and 0.44 (optimized score 0.52) for the pooled tissues. A transRate score >0.22 is generally thought to be acceptable [31,39]. More than 90% of the reads were used to assemble the transcriptomes, and 87% (brain, pooled tissues), as well as 90% (embryonic disc) of the fragments, were considered as good mappings, while only 1.6% (brain), 3.7% (embryonic disc), and 2.7% (pooled tissues) of assembled contigs had no coverage (Supplementary Table S1).

The assessment of completeness of our assemblies by the BUSCO pipeline resulted in a moderate to high percentage of complete eukaryotic orthologues (from 69.8 to 98.0% of 255 BUSCOs) but also a significantly higher percentage of putative paralogues; in the vertebrate (3354 BUSCOs) and tetrapod (5310 BUSCOs) databases, more than half of the markers were recovered completely in the brain and embryonic tissue (Table 2). The fraction of missing BUSCOs ranged between 0.8% (Eukaryota; embryonic disc) and 54.9% (Tetrapoda; pooled tissues). These BUSCO values are comparable to recent *de novo* transcriptome studies in many vertebrates [60,61,62]. BUSCO recovery rate tends to be highest when full organism and/or multiple developmental stages were used to generate the transcriptomes, compared to those assembled from specific organs or tissues [63].

Coverage of specific gene databases (*Anolis carolinensis*, *Gekko japonicus*, *Python bivittatus*) ranged between 7% and 29%, being highest for the *Gekko* reference genome (Supplementary Table S2). Up to 40,000 transcripts (22–39%) could be aligned to one of the three databases. The duplication ratio varied between 1.3 and 1.5 and was in the range reported for vertebrate transcriptomes [32,64]. Although the proportion of misassembled contigs was low (<2%), the assemblies showed only a small number of 95%-assembled genes and isoforms (Supplementary Table S2). We assume that this might reflect biological novelty in the study species rather than fragmentation of the assemblies [41]. For example, the low proportion of completeness against the three reptile gene databases contrasts with a >40% completeness score for the vertebrate gene set and could be the result of an overrepresentation of gene sets from more intensively studied lineages [65].

Alternatively, suboptimal sample quality and a resulting higher proportion of fragmented genes due to potential RNA degradation could be a reason for the relatively low number of assembled genes and lower Eukaryota BUSCO scores, especially in the pooled tissues sample.

### 3.3. Functional Annotation

**Figure 2:**
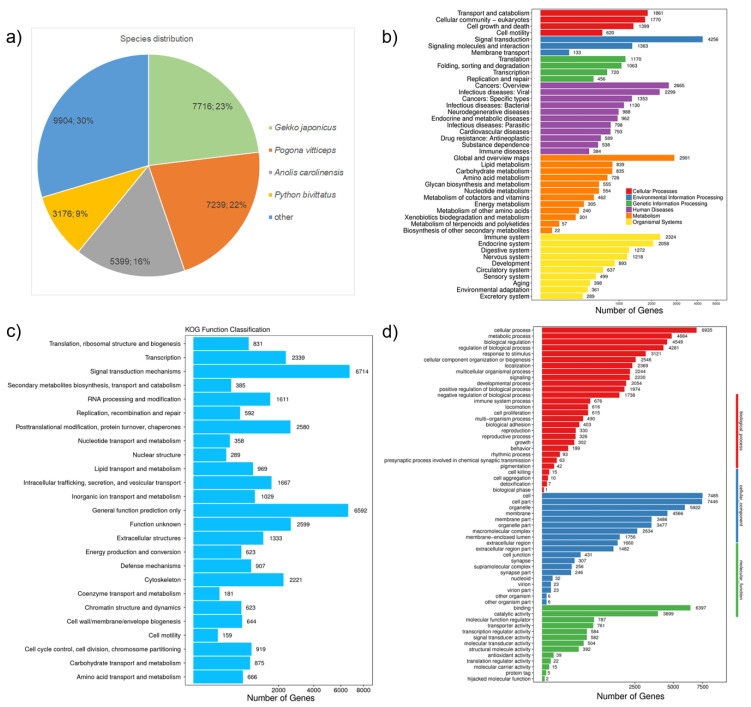
Annotation of the *Asymblepharus ladacensis* transcriptome. (**a**) Species distribution of the top BLASTx hit performed against NR database; (**b**) GO (Gene Onthology) assignments as predicted by Blast2GO; (**c**) functional distribution of KOG (EuKaryotic Orthologous Groups) annotation and (**d**) KEGG (Kyoto Encyclopedia of Genes and Genomes) classifications of assembled transcripts.

Annotation of the complete set of transcriptomes from all three samples resulted in 39,975 (51.94%) transcripts annotated in at least one of the seven databases used for functional annotation; 7292 sequences generated hits in all of these databases (Table 3). Approximately 70% of the top hits matched to genes from *Gekko japonicus* (7716; 23.07%), *Pogona vitticeps* (7239; 21.65%), *Anolis carolinensis* (5399; 16.14%), and *Python bivittatus* (3176; 9.5%), Figure 2.

In terms of the biological process ontology (GO database categories), the most common categories were cellular processes (6935), metabolic processes (4884), and biological regulation (4549). The most frequent classifications for the cellular component ontology were cellular (7485), cell compartments (7446), and organelles (5922). Regarding the molecular function, major categories involved binding (6397), catalytic activity (3899), and molecular function regulator (787) (Figure 2). The KOG functional classification revealed genes of signal transduction mechanisms (6714), general function (6592), unknown function (2599), posttranslational modification (2580), and transcription (2339) as top five categories (Figure 2).

We found 44,956 transcripts in the KEGG database that aligned to entries associated with pathways of cellular processes (5650), environmental information processing (5752), genetic information processing (3409), human diseases (12,499), metabolism (7697), and organismal systems (9949). Predominantly, the genes were enriched in “signal transduction” (4256), followed by “global and overview maps” (2901), “cancers: overview” (2665), “immune system” (2324), and “infectious diseases: viral” (2299) categories (Figure 2).

### 3.4. Positive Selection

Among the 143 PSGs previously reported for the high-elevation lineage of the toad-headed agama *Phrynocephalus vlangalii* [15], we found ten genes to be likewise under positive selection in *Asymblepharus* based on all three tests that we performed. Two of these genes (IL1RAP, GRK6) were specific to the *Asymblepharus* branch of the gene tree (Table 4 and Supplementary Table S3).

Out of the 410 transcripts reported to be under positive selection in lowland frog and lizard species [7], 321 could be identified in *Asymblepharus*. Of these, 23 (7.2%) transcripts were found to be under positive selection in the Ladakh Ground Skink in all three tests (Table 5). Similarly, a total of 32 (7.1% of 449 transcripts) PSGs of colline, 24 (7.6% of 314 transcripts) of submontane and montane, and 24 (6.9% of 350 transcripts) of subalpine and alpine frog and lizard species were tested to be under positive selection in *Asymblepharus*. Moreover, out of the 32 parallel PSGs that were identified in both high-elevation frog and lizard species [7], one gene (PGS1) showed positive selection in the Ladakh Ground Skink (Table 5). Analyzing these genes under positive selection for GO terms and pathways reveals an overrepresentation of genes involved in the following processes: low-density lipoprotein receptors and catabolic processes; mitochondrial citrate transmembrane transport; glycolysis and fructose/galactose metabolism; nucleoside phosphate binding; p53 pathway feedback loop (involved in DNA repair), platelet-derived growth (PDGF) factor binding (involved in blood-vessel formation).

## 4. Discussion

This study presents the first transcriptome sequences from different tissues of the Ladakh Ground Skink *Asymblepharus ladacensis*, a high-altitude reptile species endemic to the Greater Himalayas. We provide high-quality *de novo* transcript assemblies and well-annotated results, enabling comparisons with transcriptomes of related scincid or higher lizards available at public databases. Although squamate reptiles (lizards and snakes) represent one of the most diverse vertebrate groups with over 10,000 species spanning more than 200 million years of evolution [66], genomic data of squamates are limited and still poorly studied [67]. To our knowledge, no annotated transcriptome has been published for the genus *Asymblepharus* so far. Although our study is mostly descriptive, it has yielded discoveries with respect to genes known to play roles in the adaptation of vertebrates to high elevations and adds data resources for genomic studies in Himalayan herpetofauna.

Yang et al. [15] used comparative transcriptomic analyses of two closely related lizards, *Phrynocephalus przewalskii* from low elevations (500–1500 m a.s.l.) and *P. vlangalii* from high elevations (2000–4600 m a.s.l.), to identify candidate genes that are potentially linked to adaptation to high elevation environments. In addition, Sun et al. [7] tested amphibian and reptile populations at various altitudes in Tibet, which show parallel evolution. These studies demonstrated convergent and continuous adaptation to high elevations in Anura (Ranidae) and Sauropsida (Agamidae). Genes with related functions, especially DNA repair and energy metabolism, exhibit featured rapid changes and are positively correlated to elevation. These data let us assume that a similar genomic high-elevation selection syndrome might be detectable in *Asymblepharus*, sampled in 2714 m a.s.l. (Methods), and with a vertical distribution between ~2500 and 5500 m ([26] and references therein).

Indeed, we identified a total of 10 out of 143 and 104 out of ~1500 key genes [7,15] under positive selection in the *Asymblepharus* transcriptome. Interestingly, several of the 10 genes (Table 4) have been reported to be under positive selection or significantly enriched or differentially methylated for pathways consistent with physiological compensation for limited oxygen in high elevation dwellers, e.g., IL1RAP [68] (human), MIA3 [69] (pika), and MICU1 [70] (Ladakhi cow). Several genes we identified to be under positive selection have GO terms that suggest their involvement in, e.g., energy metabolism and DNA repair. It is well-known that high UV radiation and hypoxia are major challenges for organisms in high-altitude habitats. The extreme environments in the Greater Himalayas necessitate high energy metabolism, strong resistance to UV by an efficient DNA repair, and adaptation to hypoxia in species endemic to these mountains. However, the proportion of genes we found in *Asymblepharus* per ‘altitude-specific’ gene group does not appear to reflect a convergently evolved gene set as previously reported [7]. In other words, we found a comparable number of genes under positive selection from the group of genes identified for lowland species and those identified for colline, montane, or alpine species. Given the idea of convergent evolutionary changes and, thus, a gradual accumulation of high-elevation genetic adaptations, a higher number of candidate genes reported for montane and alpine species would have been expected in *Asymblepharus* compared to those genes reported in species distributed in lower elevations. The potential reasons for this supposed lack of confirmation of the suggested pattern are complex. A major limitation is that our analyses were restricted so far to a single *Asymblepharus* transcriptome. Due to limitations of sampling, data were unobtainable from radiation of species or an altitudinal gradient as available for frogs and other lizards [7,15], preventing us from intraspecific comparisons. Moreover, data of a single specimen cannot reflect the breadth of allelic diversity in the selected genes, putatively associated with adaptations to high altitude, especially in the view of wide vertical distribution. Another deficiency results from the fact that only one female but no male could be sequenced. Indeed, many genes that might be sex-specifically expressed might not have been sequenced or characterized with our approach. Therefore, future research with multiple high-elevation species and populations across a larger scale of altitudinal variation should validate genes known to contribute to high elevation adaptation in scincid reptiles and, thus, yield additional evidence for potential convergent evolution.

A promising future genomic approach might be to include populations of *Asymblepharus* between the species‘ lower and upper distributional periphery, sampling three populations at each of four elevation levels (e.g., <2000 m, 3000 m, 4000 m, and 5000 m a.s.l.), to investigate the expression of genes presumably related to adaptions to high altitude. It would also be desirable to reveal potential tissue-specific expression patterns across altitude-associated genes, samples of organs sensitive to UV radiation, and for oxygen, e.g., heart, lung, skin, and embryonic structures, which are particularly of interest. Moreover, to address adaptive convergence, additional comparative transcriptomic analyses on *Japalura* or *Laudakia* species might be promising since these taxa have a similarly broad vertical distribution as *Asymblepharus* and often co-occur with sympatric ground skinks. *Asymblepharus* might even become an excellent model to study local adaptation by reciprocal transplantation experiments between high and low altitude populations. Such studies could enhance our understanding of how organisms might cope with rapid environmental changes in fluctuating demographic contexts. However, such intensive field studies require adequate access to suitable habitats in the Himalayas and, thus, a much higher logistic and financial effort than available in our pilot study.

## 5. Conclusions

In summary, our present study provides the first transcriptomic data for a Himalayan reptile of the genus *Asymblepharus* and evidence for genes under positive selection for high-altitude adaptation of the Ladakh Ground Skink. Further research is encouraged to validate the key genes confirmed in this study by population genetic and functional genomic approaches. Comparative sequencing analyses for other *Asymblepharus* species may enable further insights into the adaptive basis of reptiles to different altitude environments in the Himalayas. Our data are available for future investigations on the evolution and environmental adaptation in Himalayan high-altitude vertebrates.

## Figures and Tables

**Table 1:** Summary of sequencing data used to obtain the *de novo* transcriptome assemblies of *Asymblepharus ladacensis* based on paired-end Illumina sequencing. Final assemblies based on four unique assemblies per sample generated by ORP using different assemblers and k-mers.

	Brain Tissue	Embryonic Disc Tissue	Pooled Tissue
Number of paired-end raw reads	74,780,628	73,557,120	64,195,486
Number of cleaned reads	73,139,294	71,887,892	61,397,610
Number of base pairs in final assembly	102,605,079	98,917,807	47,613,446
Number of transcripts in final assembly	151,718	105,133	66,696
Average transcript length (bp)	676	940	712
Minimum transcript length (bp)	131	131	131
Maximum transcript length (bp)	17,543	18,168	15,866
N50	1215	2052	1194
N90	257	311	278
GC% content of the final ORP assembly	0.48	0.48	0.48

**Table 2:** Benchmarking Universal Single-Copy Orthologs (BUSCO) results based on the eukaryotic (EU, eukaryota_odb10; 255 BUSCOs), vertebrates (VB, vertebrata_odb10; 3354 BUSCOs), and tetrapod databases (TP, tetrapoda_odb10, 5310 BUSCOs) searched. BUSCO searches for completed, single-copy, duplicated, fragmented, and missing orthologs within given genomes.

BUSCO Statistics	Brain			Embryonic Disc			Pooled Tissues		
	EU	VB	TP	EU	VB	TP	EU	VB	TP
Complete	220/255 (86.3%)	2052/3354 (61.1%)	2696/5310 (50.8%)	250/255 (98.0%)	2743/3354 (81.8%)	3778/5310 (71.1%)	178/255 (69.9%)	1405/3354 (41.9%)	1750/5310 (33.0%)
Single-copy	189/255 (74.1%)	1759/3354 (52.4%)	2305/5310 (43.4%)	185/255 (72.5%)	1871/3354 (55.8%)	2555/5310 (48.1%)	148/255 (58.0%)	1150/3354 (34.3%)	1421/5310 (26.8%)
Duplicated	31/255 (12.2%)	293/3354 (8.7%)	391/5310 (7.4%)	65/255 (25.5%)	872/3354 (26.0%)	1223/5310 (23.0%)	30/255 (11.8%)	255/3354 (7.6%)	329/5310 (6.2%)
Fragmented	23/255 (9.0%)	589/3354 (17.6%)	709/5310 (13.4%)	3/255 (1.2%)	221/3354 (6.6%)	323/5310 (6.1%)	47/255 (18.4%)	654/3354 (19.5%)	645/5310 (12.1%)
Missing	12/255 (4.7%)	713/3354 (21.3%)	1905/5310 (35.8%)	2/255 (0.8%)	390/3354 (11.6%)	1209/5310 (22.8%)	30/255 (11.8%)	1295/3354 (38.6%)	2915/5310 (54.9%)

**Table 3:** Number (N) of transcripts identified in *Asymblepharus ladacensis* that are shared (=Intersection) and unique among seven annotation database resources. GO—Gene Onthology; InterPro—integrative protein signature database; KEGG—Kyoto Encyclopedia of Genes and Genomes; KOG— EuKaryotic Orthologous Groups; NR—non-redundant protein database; NT—non-redundant nucleotide database; SwissProt—Swiss Protein Sequence Database.

	Total	NR	NT	SwissProt	KEGG	KOG	InterPro	GO	Intersection	Overall
N	76,968	33,444	34,114	30,994	28,961	26,608	27,013	11,010	7292	39,975
%	100	43.45	44.32	40.27	37.63	34.57	35.10	14.30	9.47	51.94

**Table 4:** Summary of the positive selection analysis for high-altitude candidate genes of a toad-headed agama (*Phrynocephalus vlangalii*) [15] tested likewise positive in *Asymblepharus* (A) using BUSTED (B); *p*-value > 0.05) [55], FUBAR (FB; number of sites under positive selection) [56] and aBSREL (aB) [57,58] methods. PSGs are represented by the last six digits of the anole lizard’s (*Anolis carolinensis*) ENSEMBL gene and transcript identifiers (starting with ENSACAG00000, or ENSACAT00000, respectively).

GeneID	Gene	*p*-Value [15]	Gene Description	Transcript	B	FB	aB
000773	IL1RAP	3.58 × 10^−2^	Interleukin 1 receptor accessory protein	000813	<0.00 × 10^−5^	2	A.
000907	MICU1	1.79 × 10^−2^	Mitochondrial calcium uptake 1	000909	1.30 × 10^−3^	1	yes
001142	TARBP1	1.39 × 10^−6^	TAR RNA binding protein 1	001104	<0.00 × 10^−5^	1	yes
002254	MIA3	1.83 × 10^−2^	Melanoma inhibitory activity family member 3	002276	1.93 × 10^−2^	1	yes
002549	RPS2	1.99 × 10^−2^	Ribosomal protein S2	002541	5.00 × 10^−4^	2	yes
002995	RNF10	4.38 × 10^−2^	Ring finger protein 10	003046	2.63 × 10^−2^	5	yes
003987	NUP107	1.54 × 10^−4^	Nucleoporin 107kDa	004158	<0.00 × 10^−5^	1	yes
006133	GRK6	1.07 × 10^−2^	G protein-coupled receptor kinase 6	006252	1.16 × 10^−2^	2	A.
007074	SMC4	1.56 × 10^−3^	Structural maintenance of chromosomes 4	007191	1.00 × 10^−4^	1	yes
015860	SH3RF1	4.55 × 10^−3^	SH3 domain containing ring finger 1	015968	8.90 × 10^−3^	1	yes

**Table 5:** Summary of the positive selection analysis for candidate genes of lineages of dicroglossid frogs and toad-headed agamas [7] identified across an elevational gradient, tested likewise positive in *Asymblepharus* (A) using BUSTED (B); *p*-value > 0.05) [55], FUBAR (FB; number of sites under positive selection) [56] and aBSREL (aB, number of branches with positive selection) [57,58] methods. Ensembl gene and transcript identifier (ENSACAG00000, ENSACAT00000) refers to *Anolis carolinensis*.

Lowland		Gene Description	Transcript	B	FB	aB
GeneID	Gene					
000146	PCSK9	Proprotein convertase subtilisin/kexin type 9	000163	4.40 × 10^−3^	1	2
000201	NPC1	NPC intracellular cholesterol transporter 1	000261	5.10 × 10^−3^	2	1
000264	LAMP1	Lysosomal associated membrane protein 1	000247	2.90 × 10^−3^	8	2
000531	SEPT12	Septin 12	000602	1.29 × 10^−2^	1	1
000768	SYK	Spleen associated tyrosine kinase	000802	<0.00 × 10^−5^	1	3
000798	WBP4	WW domain binding protein 4	000804	2.00 × 10^−2^	3	1
000955	QSOX1	Quiescin sulfhydryl oxidase 1	000962	9.20 × 10^−3^	3	1
001070	CADM1	Cell adhesion molecule 1	001188	1.00 × 10^−4^	1	1
002270	KANK1	KN motif and ankyrin repeat domains 1	002294	8.00 × 10^−4^	2	2
003015	VLDLR	Very low-density lipoprotein receptor	003096	1.66 × 10^−2^	3	1
003460	ANKRD12	Ankyrin repeat domain 12	003484	1.80 × 10^−3^	1	1
003908	MDM1	Mdm1 nuclear protein	003923	2.30 × 10^−3^	2	2
005884	CCDC66	Coiled-coil domain containing 66	025744	<0.00 × 10^−5^	1	1
006279	GLYR1	Glyoxylate reductase 1 homolog	006325	<0.00 × 10^−5^	1	3
007694	ACOX1	Acyl-CoA oxidase 1	007823	<0.00 × 10^−5^	8	1
008005	PHACTR2	Phosphatase and actin regulator 2	008047	4.30 × 10^−3^	5	1
008420	VTA1	Vesicle trafficking 1	008463	3.36 × 10^−2^	1	1
009938			010074	<0.00 × 10^−5^	6	3
013301	COL1A2	Collagen type I α 2 chain	013614	1.10 × 10^−3^	17	3
013917	MSH2	MutS homolog 2	014076	1.11 × 10^−2^	1	1
013984	LRRCC1	Leucine-rich repeat and coiled-coil centrosomal protein 1	014100	2.50 × 10^−2^	1	2
016683	TENT2	Terminal nucleotidyltransferase 2	016777	<0.00 × 10^−5^	2	2
017936	ATP6V0A1	ATPase H+ transporting V0 subunit a1	018008	3.50 × 10^−3^	1	1
Up to 2000 m						
Gene ID	Gene	Gene description	Transcript	B	FB	aB
000608	FBXL3	F-box and leucine-rich repeat protein 3	000548	3.47 × 10^−2^	2	1
000768	SYK	Spleen associated tyrosine kinas	000802	<0.00 × 10^0^	1	3
000837	KATNB1	Katanin p80 (WD repeat containing) subunit B 1	000896	4.96 × 10^−2^	1	1
000955	QSOX1	Quiescin sulfhydryl oxidase 1	000962	8.90 × 10^−3^	3	1
002090	KIAA0232	KIAA0232	002069	<0.00 × 10^0^	5	2
002091	RANBP2	RAN binding protein 2	002100	7.00 × 10^−3^	2	1
002556	SLC25A1	Solute carrier family 25 member 1	002543	5.00 × 10^−3^	2	1
002948	RSPH1	Radial spoke head component 1	002963	1.59 × 10^−2^	2	1
003975	TOGARAM1	TOG array regulator of axonemal microtubules 1	004004	<0.00 × 10^−5^	1	3
003987	NUP107	Nucleoporin 107	004158	<0.00 × 10^−5^	1	2
004938	RARS1	Arginyl-tRNA synthetase 1	005003	<0.00 × 10^−5^	3	2
005569	CCT4	Chaperonin containing TCP1 subunit 4	005683	2.00 × 10^−4^	2	1
006189	PARP1	Poly(ADP-ribose) polymerase 1	006356	4.11 × 10^−2^	4	1
006926	CTNND1	Catenin delta 1	007006	3.00 × 10^−4^	1	1
007100	ZNF277	Zinc finger protein 277	007173	1.17 × 10^−2^	3	3
007489	PRDX4	Peroxiredoxin 4	007498	2.20 × 10^−3^	1	1
007985	POLR3A	RNA polymerase III subunit A	008077	1.37 × 10^−2^	1	1
008005	PHACTR2	Phosphatase and actin regulator 2	008047	3.90 × 10^−3^	5	1
008475	SNTA1	Syntrophin α 1	008544	5.20 × 10^−3^	5	1
008991	KEAP1	Kelch-like ECH-associated protein 1	009010	2.00 × 10^−4^	3	1
009213	SWAP70	Switching B cell complex subunit SWAP70	009242	8.70 × 10^−3^	1	1
013059	JPH1	Junctophilin 1	013093	1.09 × 10^−2^	4	1
013301	COL1A2	Collagen type I α 2 chain	013614	1.10 × 10^−3^	17	3
013326	ADGRF5	Adhesion G protein-coupled receptor F5	013405	4.00 × 10^−4^	1	1
015062	COL3A1	Collagen type III α 1 chain	015539	3.00 × 10^−2^	11	4
015374	RANBP17	RAN binding protein 17	015630	7.90 × 10^−3^	3	2
015422	BAIAP2	BAR/IMD-domain-containing adaptor protein 2	015540	4.96 × 10^−2^	2	2
016662	PGS1	Phosphatidylglycerophosphate synthase 1	016740	<0.00 × 10^−5^	1	1
017208	FLOT1	Flotillin 1	017291	<0.00 × 10^−5^	4	2
017228	SLC4A1	Solute carrier family 4 member 1 (Diego blood group)	017345	4.36 × 10^−2^	2	1
017316	PTBP3	Polypyrimidine tract binding protein 3	017407	6.00 × 10^−4^	2	3
018003	FGFR1	Fibroblast growth factor receptor 1	018080	6.00 × 10^−4^	2	1
**2000–3500 m**						
Gene ID	Gene	Gene description	Transcript	B	FB	aB
000306	ZNF622	Zinc finger protein 622	000291	8.50 × 10^−3^	5	1
000907	MICU1	Mitochondrial calcium uptake 1	000909	1.30 × 10^−3^	1	1
001396	ABCC3	ATP-binding cassette subfamily C member 3	001480	<0.00 × 10^−5^	1	1
002254	MIA3	Melanoma inhibitory activity family member 3	002276	2.22 × 10^−2^	1	2
002779	CDH1	Cadherin 1	003031	1.50 × 10^−3^	1	1
004137	STARD13	StAR-related lipid transfer domain containing 13	004235	2.00 × 10^−4^	3	1
005084	COL1A1	Collagen type I α 1 chain	005298	5.00 × 10^−4^	18	2
006739	RALGAPB	Ral GTPase-activating protein, β subunit	006803	9.40 × 10^−3^	1	1
006920	NEO1	Neogenin 1	007074	<0.00 × 10^−5^	1	1
006926	CTNND1	Catenin delta 1	007006	3.00 × 10^−4^	1	1
007694	ACOX1	Acyl-CoA oxidase 1	007823	0.00 × 10^0^	8	1
007907	FLOT2	Flotillin 2	008015	0.00 × 10^0^	1	1
008206	ADGRG6	Adhesion G protein-coupled receptor G6	008405	1.21 × 10^−2^	4	1
009366	PLEKHG3	Pleckstrin homology and RhoGEF domain containing G3	026649	1.43 × 10^−2^	1	2
010640	MYLK	Myosin light chain kinase	010735	3.00 × 10^−4^	2	1
011707	FYN	FYN proto-oncogene, Src family tyrosine kinase	011760	5.00 × 10^−3^	1	1
013938	SPEG	SPEG complex locus	023342	3.99 × 10^−2^	2	2
014232	RBBP5	RB binding protein 5	014319	<0.00 × 10^−5^	5	2
014373	CD82	Tetraspanin	014458	2.92 × 10^−2^	5	1
015062	COL3A1	Collagen type III α 1 chain	015539	2.92 × 10^−2^	11	4
015121	SLC26A4	Solute carrier family 26 member 4	015204	3.02 × 10^−2^	2	1
015894	PNN	Pinin, desmosome associated protein	015973	1.56 × 10^−2^	1	1
016077			016133	1.00 × 10^−3^	4	3
017036	NIF3L1	NGG1 interacting factor 3 like 1	017110	8.50 × 10^−3^	4	1
**3500–4500 m**						
GeneID	Gene	Gene description	Transcript	B	FB	aB
000773	IL1RAP	Interleukin 1 receptor accessory protein	000813	<0.00 × 10^−5^	2	5
001545	ZCCHC8	Zinc finger CCHC-type containing 8	001573	2.78 × 10^−2^	1	2
002034	CCDC138	Coiled-coil domain containing 138	002022	4.00 × 10^−4^	1	1
003612	NR3C2	Nuclear receptor subfamily 3 group C member 2	003696	4.97 × 10^−2^	7	1
004231	COL6A3	Collagen type VI α 3 chain	004512	<0.00 × 10^−5^	6	2
005058	RPL11	Ribosomal protein L11	005076	1.67 × 10^−2^	1	1
005562	PFKM	Phosphofructokinase, muscle	005957	4.83 × 10^−2^	3	2
006776	FUS	FUS RNA-binding protein	006895	6.70 × 10^−3^	1	1
006926	CTNND1	Catenin delta 1	007006	3.00 × 10^−4^	1	1
007250	ATP11B	ATPase phospholipid transporting 11B (putative)	007392	1.40 × 10^−3^	12	1
007887	ABHD3	Abhydrolase domain containing 3, phospholipase	007896	4.00 × 10^−3^	2	2
009164	NADK2	NAD kinase 2, mitochondrial	009211	1.18 × 10^−2^	1	1
009700			009686	5.50 × 10^−3^	1	1
009800	FLNA	Filamin A	010200	0.00 × 10^0^	2	2
011843	SENP7	SUMO specific peptidase 7	011850	1.43 × 10^−2^	1	1
013301	COL1A2	Collagen type I α 2 chain	013614	1.20 × 10^−3^	17	3
013313	AGAP2	ArfGAP with GTPase domain, ankyrin repeat, PH domain 2	013455	3.40 × 10^−3^	1	1
014695	RFWD2	COP1 E3 ubiquitin ligase	014789	1.00 × 10^−3^	2	2
014919	ALDOA	Aldolase, fructose-bisphosphate A	014984	2.99 × 10^−2^	2	1
015785	CDK14	Cyclin-dependent kinase 14	015872	6.10 × 10^−3^	1	1
016662	PGS1	Phosphatidylglycerophosphate synthase 1	016740	<0.00 × 10^−5^	1	1
017054	NFIX	Nuclear factor I X	017136	9.50 × 10^−3^	1	1
017166	FARSA	Phenylalanyl-tRNA synthetase subunit α	017239	2.58 × 10^−2^	1	2
017936	ATP6V0A1	ATPase H + transporting V0 subunit a1	018008	3.50 × 10^−3^	1	1
**Frogs and lizards, common genes at similar elevation**						
Gene ID	Gene	Gene description	Transcript	B	FB	aB
016662	PGS1	Phosphatidylglycerophosphate synthase 1	016740	<0.00 × 10^−5^	1	1

## Data Availability

Data generated in this study are publicly available from the NCBI GenBank database under the Bioproject ID PRJNA750278, BioSamples SAMN20458631, SAMN20458632, and SAMN20458631. All sequence data were deposited in the NCBI Sequence Read Archive (SRA, http://www.ncbi.nlm.nih.gov/Traces/sra/; accessed on 2 August 2021) under the accession numbers SRR15283177, SRR15283178, and SRR15283179; assembled sequences were transmitted to NCBI Transcriptome Shotgun Assembly Sequence Database (TSA, http://www.ncbi.nlm.nih.gov/genbank/tsa (accessed on 2 August 2021).

## Supplementary Materials

The following are available online at https://www.mdpi.com/article/10.3390/genes12091423/s1, Figure S1: Basic statistics for the data derived from brain, embryonic disc, and pooled tissues based on the rnaQUAST report, Table S1: TransRate results for *Asymblepharus ladacensis* ORP assemblies, Table S2: Results of the quality assessment of the transcriptome assemblies from brain tissue, an embryonic disc, and pooled tissues of *Asymblepharus ladacensis* using rnaQUAST and the reference database of (a) *Anolis carolinensis*, (b) *Gekko japonicus*, and (c) *Python bivittatus*, Table S3: List of positively selected genes in *Phrynocephalus* species (according to [2]) and *Asymblepharus ladacensis*, their functional categories and summary of tests for positive selection.

## References

[1] Mittermeier R.A., Robles-Gil P., Homan M., Pilgrim J., Brooks T., Mittermeier C.G., Lamoreux J., da Fonseca G.A.B. (2004). Hotspots Revisited.

[2] Hofmann S., Stoeck M., Zheng Y., Ficetola F.G., Li J.T., Scheidt U., Schmidt J. (2017). Molecular Phylogenies Indicate a Paleo-Tibetan Origin of Himalayan Lazy Toads (*Scutiger*). Sci. Rep..

[3] Yang W., Qi Y., Fu J. (2016). Genetic signals of high-altitude adaptation in amphibians: A comparative transcriptome analysis. BMC Genet..

[4] Wang G.-D., Zhang B.-L., Zhou W.-W., Li Y.-X., Jin J.-Q., Shao Y., Yang H.-C., Liu Y.-H., Yan F., Chen H.-M. (2018). Selection and Environmental Adaptation Along a Path to Speciation in the Tibetan Frog *Nanorana parkeri*. Proc. Natl. Acad. Sci. USA.

[5] Che J., Zhou W.-W., Hu J.-S., Yan F., Papenfuss T.J., Wake D.B., Zhang Y.-P. (2010). Spiny frogs (Paini) illuminate the history of the Himalayan region and Southeast Asia. Proc. Natl. Acad. Sci. USA.

[6] Lu B., Jin H., Fu J. (2020). Molecular convergent and parallel evolution among four high-elevation anuran species from the Tibetan region. BMC Genom..

[7] Sun Y.B., Fu T.T., Jin J.Q., Murphy R.W., Hillis D.M., Zhang Y.P., Che J. (2018). Species Groups Distributed across Elevational Gradients Reveal Convergent and Continuous Genetic Adaptation to High Elevations. Proc. Natl. Acad. Sci. USA.

[8] Dorge T., Hofmann S., Wangdwei M., Duoje L., Solhøy T., Miehe G. (2007). The ecological specialist, *Thermophis baileyi* (Wall, 1907)—New records, distribution, and biogeographic conclusions. Herpetol. Bull..

[9] Jin Y.T., Brandt D.Y.C., Li J., Wo Y., Tong H., Shchur V. (2020). Elevation as a selective force on mitochondrial respiratory chain complexes of the *Phrynocephalus* lizards in the Tibetan plateau. Curr. Zool..

[10] Baig K.J., Wagner P., Ananjeva N.B., Böhme W. (2012). A morphology-based taxonomic revision of *Laudakia* Gray, 1845 (Squamata: Agamidae). Vertebr. Zool..

[11] Gangloff E.J., Telemeco R.S. (2018). High Temperature, Oxygen, and Performance: Insights from Reptiles and Amphibians. Integr. Comp. Biol..

[12] Jackson D.C. (2007). Temperature and hypoxia in ectothermic tetrapods. J. Thermal. Biol..

[13] Yang W.J., Qi Y., Bi K., Fu J. (2012). Toward Understanding the Genetic Basis of Adaption to High-Elevation Life in Poikilothermic Species: A Comparative Transcriptomic Analysis of Two Ranid Frogs, *Rana chensinensis* and *R. kukunoris*. BMC Genom..

[14] Li J.T., Gao Y.-D., Xie L., Deng C., Shi P., Guan M.-L., Huang S., Ren J.-L., Wu D.-D., Ding L. (2018). Comparative genomic investigation of high-elevation adaptation in ectothermic snakes. Proc. Natl. Acad. Sci. USA.

[15] Yang W., Qi Y., Fu J. (2014). Exploring the genetic basis of adaptation to high elevations in reptiles: A comparative transcriptome analysis of two toad-headed agamas (genus *Phrynocephalus*). PLoS ONE.

[16] Hofmann S., Kuhl H., Baniya C.B., Stock M. (2019). Multi-Tissue Transcriptomes Yield Information on High-Altitude Adaptation and Sex-Determination in *Scutiger* cf. sikimmensis. Genes.

[17] Mosbrugger V., Favre L., Müllner-Riehl A., Päckert M., Mulch A., Hoorn C., Perrigo A., Antonelli A. (2018). Cenozoic Evolution of Geo-Biodiversity in the Tibeto-Himalayan Region. Mountains, Climate and Biodiversity.

[18] Pacifici M., Foden W.B., Visconti P., Watson J.E.M., Butchart S.H.M., Kovacs K.M., Scheffers B.R., Hole D.G., Martin T.G., Akçakaya H.R. (2015). Assessing species vulnerability to climate change. Nat. Clim. Chang..

[19] Xu J., Badola R., Chettri N., Chaudhary R.P., Zomer R., Pokhrel B., Hussain S.A., Pradhan S., Pradhan R., Wester P., Mishra A., Mukherji A., Shrestha A. (2019). Sustaining Biodiversity and Ecosystem Services in the Hindu Kush Himalaya. The Hindu Kush Himalaya Assessment.

[20] Kouyoumdjian L., Gangloff E., Souchet J., Cordero G.A., Dupoué A., Aubret F. (2019). Transplanting gravid lizards to high elevation alters maternal and embryonic oxygen physiology, but not reproductive success or hatchling phenotype. J. Exp. Biol..

[21] Banasiak K.J., Xia Y., Haddad G.G. (2000). Mechanisms underlying hypoxia-induced neuronal apoptosis. Prog. Neurobiol..

[22] Bickler P.E., Donohoe P.H. (2002). Adaptive responses of vertebrate neurons to hypoxia. J. Exp. Biol..

[23] Pyron R.A., Burbrink F.T., Wiens J.J. (2013). A phylogeny and revised classification of Squamata, including 4161 species of lizards and snakes. BMC Evol. Biol..

[24] Linkem C.W., Diesmos A.C., Brown R.M. (2011). Molecular systematics of the Philippine forest skinks (Squamata: Scincidae: *Sphenomorphus*): Testing morphological hypotheses of interspecific relationships. Zool. J. Linn. Soc. Lond..

[25] Shea G.M., Greer A.E. (2002). From *Sphenomorphus* to *Lipinia*: Generic reassignment of two poorly known New Guinea skinks. J. Herpetol..

[26] Borkin L.J., Litvinchuk S.N., Melnikov D.A., Skorinov D.V., Hartmann R., Weipert J., Barclay M. (2018). Altitudinal distribution of skinks of the genus *Asymblepharus* in the Western Himalaya, India (Reptilia: Sauria: Scincidae). Biodiversität und Naturausstattung im Himalaya VI.

[27] Hartmann M., Weipert J., Weigel A. (1998). Die zoologischen Nepal-Expeditionen des Naturkundemuseums Erfurt. Veröffentlichungen des Nat. Erf..

[28] Hartmann M., Weipert J. (2015). Biodiversität und Naturausstattung im Himalaya V.

[29] Andrews S. (2010). FastQC: A Quality Control Tool for High Throughput Sequence Data. http://www.bioinformatics.babraham.ac.uk/projects/fastqc/.

[30] Kopylova E., Noe L., Touzet H. (2012). SortMeRNA: Fast and accurate filtering of ribosomal RNAs in metatranscriptomic data. Bioinformatics.

[31] Mac Manes M.D. (2018). The Oyster River Protocol: A multi assembler and Kmer approach for *de novo* transcriptome assembly. PEERJ.

[32] Hoelzer M., Marz M. (2019). *De novo* transcriptome assembly: A comprehensive cross-species comparison of short-read RNA-Seq assemblers. Gigascience.

[33] Lu B., Zeng Z., Shi T. (2013). Comparative study of *de novo* assembly and genome-guided assembly strategies for transcriptome reconstruction based on RNA-Seq. Sci. China Life Sci..

[34] Bolger A.M., Lohse M., Usadel B. (2014). Trimmomatic: A flexible trimmer for Illumina sequence data. Bioinformatics.

[35] Song L., Florea L. (2015). Rcorrector: Efficient and accurate error correction for Illumina RNA-seq reads. Gigascience.

[36] Haas B., Papanicolaou A., Yassour M., Grabherr M., Blood P., Bowden J., Couger M., Eccles D., Li B., Lieber M. (2013). *De novo* transcript sequence reconstruction from RNA-seq using the Trinity platform for reference generation and analysis. Nat. Protoc..

[37] Chikhi R., Medvedev P. (2014). Informed and automated k-mer size selection for genome assembly. Bioinformatics.

[38] Kannan S., Hui J., Mazooji K., Pachter L., Tse D. (2016). Shannon: An Information-Optimal *de novo* RNA-Seq Assembler. BioRxiv.

[39] Smith-Unna R., Boursnell C., Patro R., Hibberd J.M., Kelly S. (2016). TransRate: Reference-free quality assessment of *de novo* transcriptome assemblies. Genome Res..

[40] Seppey M., Manni M., Zdobnov E.M., Kollmar M. (2019). BUSCO: Assessing Genome Assembly and Annotation Completeness. Gene Prediction. Methods in Molecular Biology.

[41] Simao F.A., Waterhouse R.M., Ioannidis P., Kriventseva E.V., Zdobnov E.M. (2015). BUSCO: Assessing genome assembly and annotation completeness with single-copy orthologs. Bioinformatics.

[42] Bushmanova E., Antipov D., Lapidus A., Suvorov V., Prjibelski A.D. (2016). rnaQUAST: A quality assessment tool for *de novo* transcriptome assemblies. Bioinformatics.

[43] Buchfink B., Xie C., Huson D.H. (2015). Fast and sensitive protein alignment using DIAMOND. Nat. Methods.

[44] Altschul S.F., Gish W., Miller W., Myers E.W., Lipman D.J. (1990). Basic Local Aligment Search Tool. J. Mol. Biol..

[45] Conesa A., Gotz S. (2008). Blast2GO: A comprehensive suite for functional analysis in plant genomics. Int. J. Plant Genom..

[46] Quevillon E., Silventoinen V., Pillai S., Harte N., Mulder N., Apweiler R., Lopez R. (2005). InterProScan: Protein domains identifier. Nucleic Acids Res..

[47] Mistry J., Finn R.D., Eddy S.R., Bateman A., Punta M. (2013). Challenges in homology search: HMMER3 and convergent evolution of coiled-coil regions. Nucleic Acids Res..

[48] Rice P., Longden I., Bleasby A. (2000). EMBOSS: The European Molecular Biology Open Software Suite. Trends Genet..

[49] Litvinchuk S.N., Borkin L.J., Hofmann S. (2019). Rediscovery of the high-altitude Lazy Toad, *Scutiger occidentalis* Dubois, 1978, in India. Russ. J. Herpetol..

[50] Katoh K., Standley D.M. (2013). MAFFT multiple sequence alignment software version 7: Improvements in performance and usability. Mol. Biol. Evol..

[51] Price M.N., Dehal P.S., Arkin A.P. (2010). FastTree 2—Approximately maximum-likelihood trees for large alignments. PLoS ONE.

[52] Kosakovsky Pond S.L., Poon A.F.Y., Velazquez R., Weaver S., Hepler N.L., Murrell B., Shank S.D., Magalis B.R., Bouvier D., Nekrutenko A. (2020). HyPhy 2.5—A Customizable Platform for Evolutionary Hypothesis Testing Using Phylogenies. Mol. Biol. Evol..

[53] Pond S.L., Frost S.D., Muse S.V. (2005). HyPhy: Hypothesis testing using phylogenies. Bioinformatics.

[54] Spielman S.J., Weaver S., Shank S.D., Magalis B.R., Li M., Kosakovsky Pond S.L., Anisimova M. (2019). Evolution of Viral Genomes: Interplay Between Selection, Recombination, and Other Forces. Evolutionary Genomics. Methods in Molecular Biology.

[55] Murrell B., Weaver S., Smith M.D., Wertheim J.O., Murrell S., Aylward A., Eren K., Pollner T., Martin D.P., Smith D.M. (2015). Gene-wide identification of episodic selection. Mol. Biol. Evol..

[56] Murrell B., Moola S., Mabona A., Weighill T., Sheward D., Kosakovsky Pond S.L., Scheffler K. (2013). FUBAR: A fast, unconstrained Bayesian approximation for inferring selection. Mol. Biol. Evol..

[57] Smith M.D., Wertheim J.O., Weaver S., Murrell B., Scheffler K., Pond S.L.K. (2015). Less is more: An adaptive branch-site random effects model for efficient detection of episodic diversifying selection. Mol. Biol. Evol..

[58] Kosakovsky P.S.L., Murrell B., Fourment M., Frost S.D., Delport W., Scheffler K. (2011). 2011 A random effects branch-site model for detecting episodic diversifying selection. Mol. Biol. Evol..

[59] Mi H., Muruganujan A., Thomas P.D. (2013). PANTHER in 2013: Modeling the evolution of gene function, and other gene attributes, in the context of phylogenetic trees. Nucleic Acids Res..

[60] Moreno-Santillan D.D., Machain-Williams C., Hernandez-Montes G., Ortega J. (2019). *De Novo* Transcriptome Assembly and Functional Annotation in Five Species of Bats. Sci. Rep..

[61] Theissinger K., Falckenhayn C., Blande D., Toljamoc A., Gutekunstb J., Makkonenc J., Jussilac J., Lykob F., Schrimpfa A., Schulza R. (2016). *De novo* assembly and annotation of the freshwater crayfish *Astacus astacus* transcriptome. Mar. Genom..

[62] Waits D.S., Simpson D.Y., Sparkman A.M., Bronikowski A.M., Schwartz T.S. (2020). The utility of reptile blood transcriptomes in molecular ecology. Mol. Ecol. Resour..

[63] Carruthers M., Yurchenko A.A., Augley J.J., Adams C.E., Herzyk P., Elmer K.R. (2018). *De novo* transcriptome assembly, annotation and comparison of four ecological and evolutionary model salmonid fish species. BMC Genom..

[64] Bushmanova E., Antipov D., Lapidus A., Prjibelski A.D. (2019). rnaSPAdes: A *de novo* transcriptome assembler and its application to RNA-Seq data. Gigascience.

[65] Gates K., Sandoval-Castillo J., Bernatchez L., Beheregaray L.B. (2017). *De novo* transcriptome assembly and annotation for the desert rainbowfish (*Melanotaenia splendida tatei*) with comparison with candidate genes for future climates. Mar. Genom..

[66] Murphy W.J., Pringle T.H., Crider T.A., Springer M.S., Miller W. (2007). Using genomic data to unravel the root of the placental mammal phylogeny. Genome Res..

[67] Pasquesi G.I.M., Adams R.H., Card D.C., Schield D.R., Corbin A.B., Perry B.W., Reyes-Velasco J., Ruggiero R.P., Vandewege M.W., Shortt J.A. (2018). Squamate reptiles challenge paradigms of genomic repeat element evolution set by birds and mammals. Nat. Commun..

[68] Childebayeva A., Goodrich J.M., Léon-Velarde F., Rivera-Chira M., Kiyamu M., Brutsaert T., Dolinoy D., Bigham A. (2021). Genome-wide DNA methylation changes associated with high-altitude acclimatization during an Everest base camp trek. Front. Physiol..

[69] Solari K.A., Ramakrishnan U., Hadly E.A. (2018). Gene expression is implicated in the ability of pikas to occupy Himalayan elevational gradient. PLoS ONE.

[70] Verma P., Sharma A., Sodhi M., Thakur K., Kataria R., Niranjan S.K., Bharti V., Kumar P., Giri A., Kalia S. (2018). Transcriptome analysis of circulating PBMCs to understand mechanism of high-altitude adaptation in native cattle of Ladakh region. Sci. Rep..

