# Supplementary material for: De novo Assembly, Annotation, and Analysis of Transcriptome Data of the Ladakh Ground Skink Provide Genetic Information on High-Altitude Adaptation
Source: Genes (Basel). 2021 Sep 16;12(9):1423. doi: 10.3390/genes12091423 (PMC8466045; doi:10.3390/genes12091423)
Supplement: Supplementary file 1 [file genes-12-01423-s001.zip › genes-1356036-supplementary.pdf]

Supplementary Material

De novo assembly, annotation, and analysis of transcriptome data of the Ladakh ground skink provide genetic information on high-altitude adaptation

Sylvia Hofmann, Chitra Bahadur Baniya, Matthias Stöck, Lars Podsiadlowski

Table S1: TransRate results for *Asymblepharus ladacensis* ORP assemblies.

Contig and read mapping metrics	Brain tissue	Embryonic disc	Pooled tissues
n_seqs (number of contigs in the assembly)	151,718	105,133	66,696
smallest (size of the smallest contig)	131	131	131
largest (size of the largest contig)	17,543	18,168	15,866
n_bases (number of bases included in the assembly)	102,605,079	98,917,807	47,613446
mean_len (mean length of the contigs)	675.79	940.28	712.40
n_under_200 (number of contigs shorter than 200 bases)	433	364	562
n_over_1k (number of contigs greater than 1,000 bases long)	25,086	27,175	12,828
n_over_10k (number of contigs greater than 10,000 bases long)	30	97	5
n_with_orf (number of contigs that had an open reading frame)	24,080	29,622	14,892
mean_orf_percent (mean % of the contig covered by the ORF)	50.48	53.95	54.65
n90	257	311	278
n70	523	998	594
n50	1,215	2,052	1,194
n30	2,369	3,364	2,120
n10	4,358	5,704	3,795
gc (% of bases that are G or C)	0.48	0.48	0.48
bases_n (number of bases that are N)	59,950	34,430	5,930
proportion_n (proportion of bases that are N)	0.00058	0.00035	0.00012
fragments (number of read pairs provided)	36,569,647	35,943,946	30,698,805
fragments_mapped (total number of read pairs mapping)	34,060,305	34,203,135	28,733,111
p_fragments_mapped (proportion of read pairs mapping)	0.93	0.95	0.94
good_mappings (number of read pairs mapping indicative of good assembly)	31,630,550	32,201,894	26,792,615
p_good_mapping (proportion of read pairs mapping indicative of good assembly)	0.87	0.90	0.87
bad_mappings (number of reads pairs mapping indicative of bad assembly)	2,429,755	2,001,241	1,940,496
potential_bridges (number of potential links between contigs supported by reads)	33,917	22,237	12,252
bases_uncovered (number of bases that are not covered by any reads)	3,135,766	5,843,986	1,972,391
p_bases_uncovered (proportion of bases that are not covered by any reads)	0.030	0.059	0.041
contigs_uncovbase (number of contigs with at least one base without read coverage)	46,397	41,659	22,234
p_contigs_uncovbase (proportion of contigs with at least one base without read coverage)	0.306	0.396	0.333
contigs_uncovered (number of contigs with a mean per-base read coverage < 1)	2,400	3,882	1,821
p_contigs_uncovered (proportion of contigs with a mean per-base read coverage < 1)	0.016	0.037	0.027
contigs_lowcovered (number of contigs with a mean per-base read coverage < 10)	80,646	64,971	44,180

p_contigs_lowcovered (proportion of contigs with a mean per-base read coverage < 10)	0.532	0.618	0.662
contigs_segmented (number of contigs with ≥50% chance of being segmented)	17,573	9,783	5,062
p_contigs_segmented (proportion of contigs with ≥50% chance of being segmented)	0.116	0.093	0.076
transRate assembly score	0.444	0.452	0.436
transRate assembly optimal_score	0.494	0.557	0.524
transRate optimal cutoff	0.336	0.373	0.455
weighted	3.198	5.074	6.414

Table S2. Results of the quality assessment of the transcriptome assemblies from brain tissue, an embryonic disc, and pooled tissues of *Asymblepharus ladacensis* using rnaQUAST and the reference database of **a)** *Anolis carolinensis*, **b)** *Gekko japonicus*, and **c)** *Python bivittatus*. Assembly quality was evaluated in terms of the alignability, accuracy, completeness/sensitivity, specificity, continuity, and misassembly. For details on metrics see the rnaQUAST manual on https://github.com/ablab/rnaquast and Bushmanova et al. [1].

**a)**	*ORP assembly (brain)*	*ORP assembly (disc)*	*ORP assembly (pooled)*
Basic metrics of database and transcripts			
Number of genes in the database	21,865	21,865	21,865
Avg. number of exons per isoform from the database	11.712	11.712	11.712
Number of assembled transcripts	151,718	105,133	66,696
Number of transcripts > 500 bp	48,884	44,358	25,772
Number of transcripts > 1000 bp	25,112	27,196	12,848
Alignment metrics			
Number of aligned transcripts	33,697 (22.21%)	34,931 (33.23%)	22,704 (34.04%)
Avg. aligned fraction for a transcript	0.640	0.609	0.692
Avg. alignment length (bp)	627.346	794.072	549.530
Accuracy			
Avg. mismatches per transcript (bp)	34.363	35.427	34.332
Completeness (sensitivity)			
Database coverage	0.117	0.132	0.071
Mean isoform coverage	0.357	0.394	0.344
Duplication ratio	1.307	1.493	1.324
Number of > 50% covered genes	3,395	3,939	2,229
Number of > 95% covered genes	112	136	60
Number of > 50% covered isoforms	3,540	4,225	2,319
Number of > 95% covered isoforms	112	145	61
Specificity			
Number of > 50% matched transcripts	18,963	18,136	15,053
Number of > 95% matched transcripts	4,702	3,757	4,366
Mean fraction of transcript matched	0.584	0.565	0.654
Number of unannotated transcripts	2,117	1,468	898
Continuity			
Number of > 50% assembled genes	2,735	3,413	1,681
Number of > 95% assembled genes	88	100	44
Number of > 50% assembled isoforms	2,828	3,663	1,749
Number of > 95% assembled isoforms	88	109	45
Mean isoform assembly	0.312	0.358	0.290
Misassemblies			
Number of misassemblies	1,124 (0.74%)	1,793 (1.71%)	563 (0.84%)

**b)**	*ORP assembly (brain)*	*ORP assembly (disc)*	*ORP assembly (pooled)*
Basic metrics of database and transcripts			
Number of genes in the database	21,217	21,217	21,217
Avg. number of exons per isoform from the database	9.741	9.741	9.741
Number of assembled transcripts	151,718	105,133	66,696
Number of transcripts > 500 bp	48,884	44,358	25,772
Number of transcripts > 1000 bp	25,112	27,196	12,848
Alignment metrics			
Number of aligned transcripts	38,930 (25.66%)	39,535 (37.60%)	25,939 (38.89%)
Avg. aligned fraction for a transcript	0.646	0.624	0.697
Avg. alignment length (bp)	616.084	792.887	547.690
Accuracy			
Avg. mismatches per transcript (bp)	34.365	36.417	34.279

Completeness / sensitivity			
Database coverage	0.258	0.293	0.162
Mean isoform coverage	0.469	0.510	0.411
Duplication ratio	1.346	1.542	1.345
Number of > 50% covered genes	5,582	6,358	3,305
Number of > 95% covered genes	473	574	169
Number of > 50% covered isoforms	5,709	6,639	3,404
Number of > 95% covered isoforms	475	588	176
Specificity			
Number of > 50% matched transcripts	20,393	19,452	16,475
Number of > 95% matched transcripts	4,949	4,025	4,776
Mean fraction of transcript matched	0.54	0.535	0.626
Number of unannotated transcripts	5,061	3,775	2,215
Continuity			
Number of > 50% assembled genes	4,348	5,418	2,387
Number of > 95% assembled genes	365	426	123
Number of > 50% assembled isoforms	4,431	5,668	2,471
Number of > 95% assembled isoforms	365	435	129
Mean isoform assembly	0.404	0.461	0.344
Misassemblies			
Number of misassemblies	1,167 (0.77%)	2,022 (1.92%)	585 (0.88%)

**c)**	*ORP assembly (brain)*	*ORP assembly (disc)*	*ORP assembly (pooled)*
Basic metrics of database and transcripts			
Number of genes in the database	21,972	21,972	21,972
Avg. number of exons per isoform from the database	11.028	11.028	11.028
Number of assembled transcripts	151,718	105,133	66,696
Transcripts > 500 bp	48,884	44,358	25,772
Transcripts > 1000 bp	25,112	27,196	12,848
Alignment metrics			
Number of aligned transcripts	33,954 (22.38%)	35,225 (33.51%)	22,949 (34.41%)
Avg. aligned fraction for a transcript	0.633	0.596	0.687
Avg. alignment length (bp)	618.003	775.568	544.361
Accuracy			
Avg. mismatches per transcript (bp)	38.326	40.540	36.932
Completeness / sensitivity			
Database coverage	0.133	0.150	0.083
Mean isoform coverage	0.378	0.406	0.342
Duplication ratio	1.314	1.490	1.321
Number of > 50% covered genes	3,792	4,202	2,342
Number of > 95% covered genes	261	258	88
Number of > 50% covered isoforms	3,913	4,452	2,443
Number of > 95% covered isoforms	266	270	94
Specificity			
Number of > 50% matched transcripts	18,444	17,296	14,913
Number of > 95% matched transcripts	4,352	3,407	4,182
Mean fraction of transcript matched	0.569	0.548	0.648
Number of unannotated transcripts	2,474	1,796	992
Continuity			
Number of > 50% assembled genes	3,025	3,648	1,764
Number of > 95% assembled genes	201	202	65
Number of > 50% assembled isoforms	3,110	3,877	1,844
Number of > 95% assembled isoforms	204	212	71
Mean isoform assembly	0.331	0.371	0.291
Misassemblies			
Number of misassemblies	1,220 (0.80%)	2,052 (1.95%)	629 (0.94%)

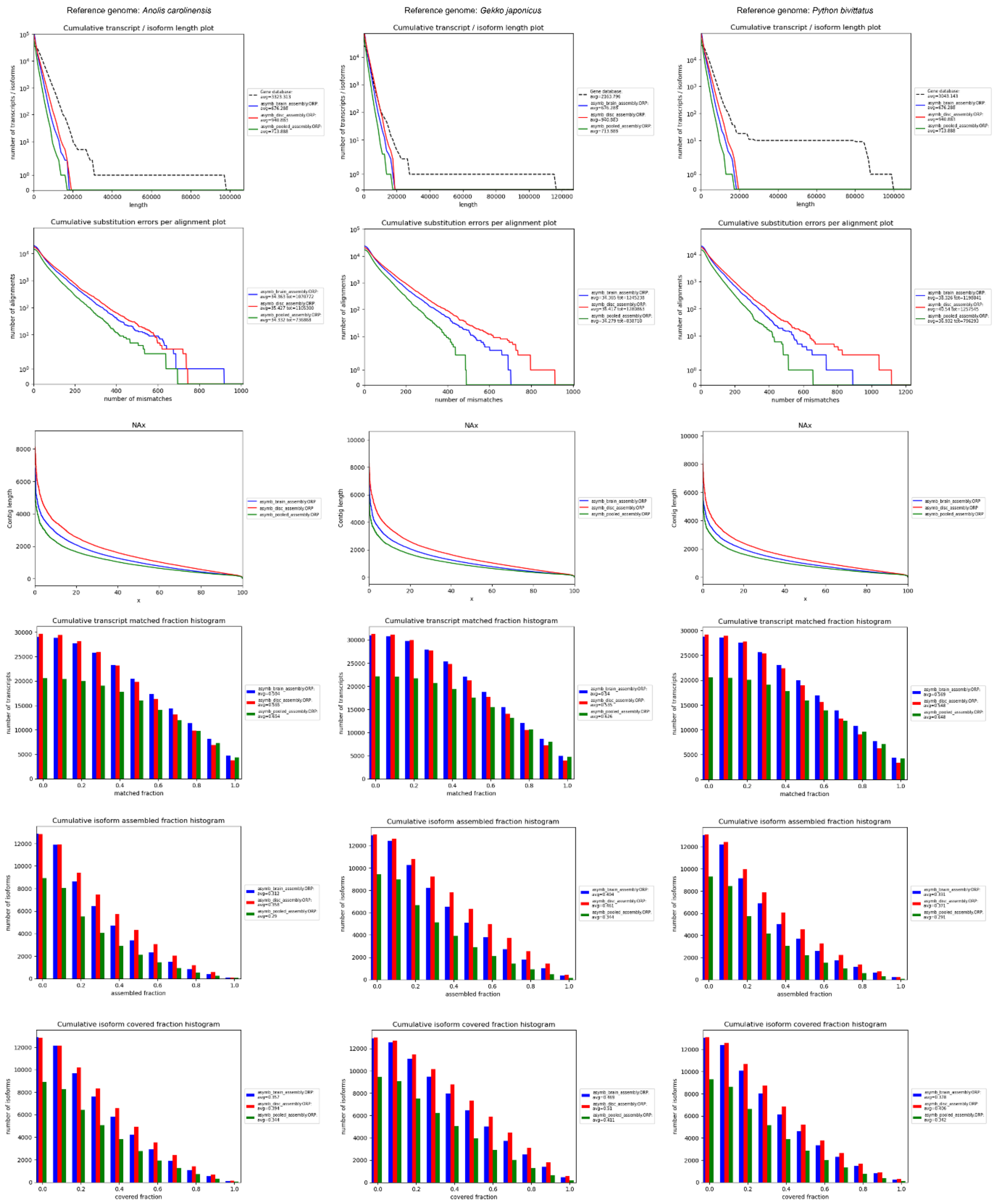

Figure S1. Basic statistics for the data derived from brain, embryonic disc, and pooled tissues based on the rnaQUAST report (from upper to lower line): transcript lengths; mismatch rates; Nx plot for transcripts (Nx is a maximal number N, such that the total length of all transcripts longer than N bp is at least x% of the total length of all transcripts); number of transcript alignments per isoform of the reference database; number of isoforms of the reference database that have at least x% captured by a single assembled transcript; number of isoforms of the reference database that have at least x% of bases covered by all alignments.

Table S3. List of positively selected genes in *Phrynocephalus* species (according to [2]) and *Asymblepharus ladacensis*, their functional categories and summary of tests for positive selection. The 10 genes that were found to be under positive selection based on all three tests are indicated bold, and among them the two genes with the *Asymblepharus* branch of the gene tree under selection are indicated bold italic. BUSTED [3] = gene-wide test for positive selection; FUBAR [4] = site-level positive selection; aBSREL [5,6] = branch test for positive selection (indicated here if the *Asymblepharus* branch of the gene tree was found to be under selection).

Anolis Gene	*Gene symbol*	*P-value* [2]	*GO category*	*Category w acc. Evolution*	*Anolis gene transcript this study*	*BUSTED p-value*	*FUBAR*	*aBSREL*
ENSACAG: 000155	CHEK1	5.81E-03		protein serine/threonine kinase activity	ENSACAT: 000155	5.00E-01	0	no
ENSACAG: 000535	CLN5	2.04E-02		integral to membrane	ENSACAT: 000511	4.18E-01	1	no
ENSACAG: 000600	-	1.13E-03			ENSACAT: 000724	4.14E-01	2	yes
ENSACAG: 000644	PROS1	4.27E-02		calcium ion binding	ENSACAT: 000780	1.58E-01	2	no
ENSACAG: 000798	WBP4	1.04E-02	RNA metabolic process	plasma membrane	ENSACAT: 000804	2.00E-02	3	yes
ENSACAG: 000773	***IL1RAP***	***3.58E-02***		***integral to membrane***	***ENSACAT: 000813***	***0.00E+00***	***2***	***Asymblepharus***
ENSACAG: 000682	PUM1	9.42E-04			ENSACAT: 000846	4.30E-02	1	yes
**ENSACAG: 000907**	**MICU1**	**1.79E-02**	**metal ion binding**	**calcium ion binding**	**ENSACAT: 000909**	**1.30E-03**	**1**	**yes**
ENSACAG: 000804	DARS	6.87E-03	RNA metabolic process		ENSACAT: 000981	5.00E-01	2	no
ENSACAG: 001054	-	5.22E-03			ENSACAT: 001066	4.72E-01	0	yes
ENSACAG: 001169	TMEM119	2.98E-02			ENSACAT: 001085	5.00E-01	0	no
**ENSACAG: 001142**	**TARBP1**	**1.39E-06**	**RNA metabolic process**		**ENSACAT: 001104**	**0.00E+00**	**1**	**yes**
ENSACAG: 001240	STK40	7.92E-03	cellular protein metabolic process	protein serine/threonine kinase activity	ENSACAT: 001278	4.73E-01	0	no
ENSACAG: 001269	H2AFY	2.36E-04			ENSACAT: 001295	7.00E-02	1	yes
ENSACAG: 001224	PPIL2	5.99E-03	cellular protein metabolic process		ENSACAT: 001311	5.00E-01	0	yes
ENSACAG: 001336	ATF6	2.06E-02		DNA-dependent regulation of transcription	ENSACAT: 001319	5.00E-01	2	no
ENSACAG: 001192	FARP2	2.04E-03		phospholipid binding	ENSACAT: 001324	4.95E-01	0	yes
ENSACAG: 001431	FAM19A2	4.68E-03			ENSACAT: 001369	5.00E-01	1	no
ENSACAG: 001393	-	8.83E-03			ENSACAT: 001437	5.00E-01	0	yes
ENSACAG: 001515	FRMPD4	3.26E-03			ENSACAT: 001504	5.00E-01	1	no
ENSACAG: 001356	ADAM17	4.63E-02	cellular protein metabolic process	response to hypoia	ENSACAT: 001535	5.00E-01	5	yes
ENSACAG: 001582	KLHDC10	3.77E-03			ENSACAT: 001558	5.00E-01	3	yes
ENSACAG: 001596	MRPL3	4.23E-02	gene epression		ENSACAT: 001612	5.00E-01	0	no
ENSACAG: 001614	SLC27A6	3.56E-02			ENSACAT: 001633	5.00E-01	2	yes
ENSACAG: 001733	-	4.69E-02			ENSACAT: 001718	1.11E-02	1	no
ENSACAG: 001846	SH3RF3	2.03E-02			ENSACAT: 001876	5.94E-02	5	*Asymblepharus*
ENSACAG: 001817	SF3B3	1.07E-02			ENSACAT: 001909	1.00E-04	0	yes
ENSACAG: 001956	FAM190B	5.52E-06			ENSACAT: 001934	5.00E-01	2	no
ENSACAG: 001907	MPHOSPH8	1.75E-02		plasma membrane	ENSACAT: 002024	1.21E-01	0	yes
ENSACAG: 002117	GPR63	1.73E-02		integral to membrane	ENSACAT: 002050	1.54E-01	0	yes
ENSACAG: 002035	UCHL3	9.60E-04	cellular protein metabolic process	ubiquitin thiolesterase activity	ENSACAT: 002086	2.09E-01	0	yes

ENSACAG: 002268	MAML3	9.33E-04			ENSACAT: 002224	1.89E-01	0	yes
ENSACAG: 001975	TECPR1	1.36E-02		integral to membrane	ENSACAT: 002254	1.15E-01	0	yes
ENSACAG: 001995	PTPRG	2.07E-02	cellular protein metabolic process		ENSACAT: 002272	5.00E-01	1	no
**ENSACAG: 002254**	**MIA3**	**1.83E-02**		**integral to membrane**	**ENSACAT: 002276**	**1.93E-02**	**1**	**yes**
ENSACAG: 002375	TMEM66	4.54E-02			ENSACAT: 002316	5.00E-01	1	no
ENSACAG: 002222	DAAM2	7.36E-07			ENSACAT: 002320	5.00E-01	2	no
ENSACAG: 002398	ACMSD	1.68E-04			ENSACAT: 002391	2.57E-01	0	no
ENSACAG: 002301	CLTCL1	1.92E-02			ENSACAT: 002499	4.54E-01	1	no
ENSACAG: 002559	TUBA3D	1.20E-05			ENSACAT: 002530	5.00E-01	0	yes
**ENSACAG: 002549**	**RPS2**	**1.99E-02**			**ENSACAT: 002541**	**5.00E-04**	**2**	**yes**
ENSACAG: 002879	FAM160A1	3.89E-03			ENSACAT: 002906	5.00E-01	2	no
ENSACAG: 003045	SACS	9.58E-04		etracellular region	ENSACAT: 003017	5.00E-01	1	no
**ENSACAG: 002995**	**RNF10**	**4.38E-02**	**metal ion binding**	**DNA-dependent positive regulation of transcription**	**ENSACAT: 003046**	**2.63E-02**	**5**	**yes**
ENSACAG: 003086	PRICKLE1	7.33E-03	metal ion binding		ENSACAT: 003106	5.00E-01	0	no
ENSACAG: 003237	PPP2CB	5.16E-06	cellular protein metabolic process		ENSACAT: 003250	4.70E-01	0	no
ENSACAG: 003307	-	1.30E-02			ENSACAT: 003399	4.30E-03	0	yes
ENSACAG: 003477	LEMD3	3.12E-02		integral to membrane	ENSACAT: 003468	6.60E-03	0	*Asymblepharus*
ENSACAG: 003512	OTUD4	2.26E-02			ENSACAT: 003512	5.00E-01	2	no
ENSACAG: 003566	ANLN	4.33E-02		phospholipid binding	ENSACAT: 003577	3.37E-01	7	no
ENSACAG: 003568	YARS	4.91E-02	RNA metabolic process		ENSACAT: 003627	5.00E-01	3	no
ENSACAG: 003496	ERBB2	9.24E-03	cellular protein metabolic process	apical plasma membrane;heart development	ENSACAT: 003733	1.98E-01	4	no
ENSACAG: 003893	TMEM177	1.91E-02			ENSACAT: 003870	5.36E-02	2	no
ENSACAG: 003932	KIAA1009	1.06E-02		plasma membrane	ENSACAT: 003931	1.82E-02	0	yes
**ENSACAG: 003987**	**NUP107**	**1.54E-04**		**transport**	**ENSACAT: 004158**	**0.00E+00**	**1**	**yes**
ENSACAG: 004104	SLC7A1	6.79E-03		integral to membrane	ENSACAT: 004250	5.00E-01	2	no
ENSACAG: 004142	UHRF2	1.43E-03			ENSACAT: 004348	2.60E-03	0	yes
ENSACAG: 004376	DUSP16	4.47E-04			ENSACAT: 004405	5.00E-01	0	no
ENSACAG: 004479	RAB11A	1.97E-02		plasma membrane	ENSACAT: 004493	5.00E-01	0	no
ENSACAG: 004456	WDFY1	1.09E-02	metal ion binding		ENSACAT: 004540	4.31E-01	1	no
ENSACAG: 004655	MKI67IP	4.13E-02			ENSACAT: 004636	5.00E-01	0	no
ENSACAG: 004722	-	7.53E-03	metal ion binding		ENSACAT: 004711	5.00E-01	0	no
ENSACAG: 004830	TMEM248	1.92E-04			ENSACAT: 004824	5.00E-01	0	no
ENSACAG: 004868	GMIP	1.18E-02			ENSACAT: 004879	4.50E-01	0	no
ENSACAG: 004932	SH3PXD2A	4.08E-02			ENSACAT: 004931	2.96E-01	1	no
ENSACAG: 005155	PLEKHD1	4.43E-02		phospholipid binding	ENSACAT: 005141	4.50E-01	0	no
ENSACAG: 005119	KATNAL1	2.14E-02			ENSACAT: 005153	5.00E-01	0	no
ENSACAG: 005554	PXDN	4.19E-05	metal ion binding	etracellular region	ENSACAT: 005644	0.00E+00	0	yes
ENSACAG: 005957	NR2E1	7.90E-03	gene epression	DNA-dependent regulation of transcription	ENSACAT: 005982	5.00E-01	0	no

ENSACAG: 005880	HNRPLL	1.96E-06	RNA metabolic process	mRNA binding	ENSACAT: 005999	5.00E-01	1	no
ENSACAG: 006133	***GRK6***	***1.07E-02***	***cellular protein metabolic process***	***protein serine/threonine kinase activity***	***ENSACAT: 006252***	***1.16E-02***	***2***	***Asymblepharus***
ENSACAG: 006230	NUP85	8.44E-03			ENSACAT: 006268	1.73E-01	0	yes
ENSACAG: 006278	C4ORF33	7.85E-03			ENSACAT: 006270	5.00E-01	0	no
ENSACAG: 006180	ATP10B	3.98E-02		integral to membrane	ENSACAT: 006285	5.00E-01	2	no
ENSACAG: 006237	SOS1	1.92E-02		phospholipid binding	ENSACAT: 006394	4.16E-01	1	no
ENSACAG: 006449	THOC3	1.11E-02			ENSACAT: 006452	5.00E-01	0	no
ENSACAG: 006437	PAQR3	7.40E-03		integral to membrane	ENSACAT: 006467	5.00E-01	0	no
ENSACAG: 006547	KLHL30	1.14E-04			ENSACAT: 006544	5.00E-01	0	no
ENSACAG: 006316	MYO1B	1.05E-04		plasma membrane	ENSACAT: 006705	1.31E-02	6	no
ENSACAG: 006939	NISCH	4.35E-02		plasma membrane	ENSACAT: 006970	4.81E-01	1	yes
ENSACAG: 006941	MYOF	1.15E-05		integral to membrane	ENSACAT: 006992	2.16E-01	1	no
ENSACAG: 007102	PGM2	7.30E-03	metal ion binding		ENSACAT: 007172	4.94E-01	0	yes
**ENSACAG: 007074**	**SMC4**	**1.56E-03**			**ENSACAT: 007191**	**1.00E-04**	**1**	**yes**
ENSACAG: 007063	PDK3	9.32E-04	cellular protein metabolic process		ENSACAT: 007206	5.00E-01	2	no
ENSACAG: 007393	CDKN2C	4.97E-03		negative regulation of cell proliferation	ENSACAT: 007389	4.11E-01	0	no
ENSACAG: 007503	TM4SF18	7.35E-03		integral to membrane	ENSACAT: 007499	5.00E-01	0	no
ENSACAG: 008363	MYO3A	4.80E-02	cellular protein metabolic process	protein serine/threonine kinase activity	ENSACAT: 008486	5.00E-01	0	no
ENSACAG: 008458	POLK	3.39E-05	cellular macromolecule metabolic process		ENSACAT: 008529	3.89E-02	0	yes
ENSACAG: 008580	RBM5	9.03E-03	metal ion binding	positive regulation of apoptotic process	ENSACAT: 008702	4.61E-01	2	no
ENSACAG: 008995	UBR5	3.10E-05			ENSACAT: 009154	4.52E-01	0	no
ENSACAG: 009182	CRYBG3	1.35E-07			ENSACAT: 009220	2.73E-01	0	yes
ENSACAG: 008984	ACACB	4.77E-02			ENSACAT: 009398	5.00E-01	1	no
ENSACAG: 009435	FAM98A	4.52E-02			ENSACAT: 009471	5.00E-01	1	no
ENSACAG: 009401	ARHGEF6	4.45E-02		phospholipid binding	ENSACAT: 009527	5.00E-01	0	no
ENSACAG: 009670	CCDC88C	3.33E-02			ENSACAT: 009795	3.31E-01	0	no
ENSACAG: 009819	-	6.97E-03			ENSACAT: 009824	3.32E-02	0	yes
ENSACAG: 009923	MEF2A	1.75E-03	gene epression	DNA-dependent regulation of transcription	ENSACAT: 009975	8.73E-02	2	yes
ENSACAG: 009939	USP54	2.45E-03	cellular protein metabolic process	ubiquitin thiolesterase activity	ENSACAT: 009984	5.00E-01	4	no
ENSACAG: 010109	-	3.30E-03		DNA-dependent regulation of transcription	ENSACAT: 010194	5.00E-01	1	no
ENSACAG: 010153	EGF	4.37E-02	cellular protein metabolic process	integral to membrane	ENSACAT: 010231	4.39E-01	0	no
ENSACAG: 010492	PCYT2	3.48E-02			ENSACAT: 010527	5.00E-01	0	yes
ENSACAG: 010758	SLC39A14	1.83E-02			ENSACAT: 010793	5.00E-01	0	yes
ENSACAG: 010692	APOB	2.99E-03		phospholipid binding	ENSACAT: 010830	0.00E+00	0	*Asymblepharus*
ENSACAG: 011183	MGAT4C	2.79E-02			ENSACAT: 011182	5.00E-01	0	no
ENSACAG: 011619	RAPGEF6	4.37E-04		plasma membrane	ENSACAT: 012152	2.89E-01	2	no
ENSACAG: 012174	HDAC2	2.74E-02	gene epression	DNA-dependent regulation of transcription	ENSACAT: 012291	4.73E-01	0	no
ENSACAG: 012469	ALG8	3.08E-02			ENSACAT: 012465	4.01E-01	0	yes

ENSACAG: 012450	EEA1	5.69E-03	metal ion binding	plasma membrane	ENSACAT: 012558	5.00E-01	0	no
ENSACAG: 012692	MGAT4A	3.92E-03	metal ion binding	integral to membrane	ENSACAT: 012818	5.00E-01	1	yes
ENSACAG: 013074	SCAI	4.41E-02		negative regulation of cell migration	ENSACAT: 013092	5.00E-01	0	no
ENSACAG: 013657	FAM82A2	1.61E-02			ENSACAT: 013745	5.00E-01	5	no
ENSACAG: 014430	SACM1L	1.96E-03			ENSACAT: 014546	4.31E-01	2	no
ENSACAG: 014501	WIPI2	3.37E-02			ENSACAT: 014619	3.03E-02	3	no
ENSACAG: 014727	SUV420H1	1.75E-04			ENSACAT: 014766	4.00E-01	2	no
ENSACAG: 015060	RTN1	1.65E-02		neuron differentiation	ENSACAT: 015129	5.00E-01	0	no
ENSACAG: 015082	OGDHL	1.44E-06			ENSACAT: 015266	5.00E-01	0	no
ENSACAG: 015290	MTERFD3	1.90E-02		DNA-dependent regulation of transcription	ENSACAT: 015311	5.00E-01	0	no
ENSACAG: 015407	CDHR1	7.11E-04	metal ion binding	integral to membrane	ENSACAT: 015466	3.00E-01	0	yes
ENSACAG: 015349	RABEP1	3.36E-02		protein homodimerization activity	ENSACAT: 015473	5.00E-01	1	no
ENSACAG: 015354	FNDC3A	9.92E-03			ENSACAT: 015531	5.00E-01	4	yes
ENSACAG: 015436	DNM2	3.25E-02			ENSACAT: 015615	1.86E-01	0	yes
ENSACAG: 015570	INPPL1	4.18E-02		plasma membrane	ENSACAT: 015676	5.00E-01	0	no
**ENSACAG: 015860**	**SH3RF1**	**4.55E-03**	**metal ion binding**		**ENSACAT: 015968**	**8.90E-03**	**1**	**yes**
ENSACAG: 015951	CSE1L	3.82E-02			ENSACAT: 016050	5.00E-01	0	yes
ENSACAG: 016190	HSP90B1	2.39E-02	cellular protein metabolic process	response to hypoia	ENSACAT: 016310	5.00E-01	1	no
ENSACAG: 016343	VSX2	4.82E-04	gene epression	DNA-dependent regulation of transcription	ENSACAT: 016382	5.00E-01	0	no
ENSACAG: 016403	CSPP1	1.62E-03			ENSACAT: 016444	1.25E-01	1	no
ENSACAG: 016394	ADCY9	1.20E-02	metal ion binding		ENSACAT: 016448	4.74E-01	3	*Asymblepharus*
ENSACAG: 016429	ECT2	1.20E-06		regulation of Rho protein signal transduction	ENSACAT: 016520	5.00E-01	4	no
ENSACAG: 000804	DARS	6.87E-03	RNA metabolic process		ENSACAT: 029206	5.00E-01	2	yes
ENSACAG: 002398	ACMSD	1.68E-04			ENSACAT: 029271	5.00E-01	0	no
ENSACAG: 001614	SLC27A6	3.56E-02			ENSACAT: 029294	5.00E-01	2	yes
ENSACAG: 016403	CSPP1	1.62E-03			ENSACAT: 029470	4.96E-02	1	no
ENSACAG: 003237	PPP2CB	5.16E-06	cellular protein metabolic process		ENSACAT: 029650	5.00E-01	0	no
ENSACAG: 010692	APOB	2.99E-03		phospholipid binding	ENSACAT: 029843	0.00E+00	0	*Asymblepharus*
ENSACAG: 004722	-	7.53E-03	metal ion binding		ENSACAT: 029919	4.93E-01	1	no
-	-	-			ENSACAT: 030082	5.00E-01	0	no
ENSACAG: 006939	NISCH	4.35E-02		plasma membrane	ENSACAT: 030449	5.00E-01	1	yes
ENSACAG: 009670	CCDC88C	3.33E-02			ENSACAT: 030578	3.78E-01	0	no
ENSACAG: 015082	OGDHL	1.44E-06			ENSACAT: 030647	5.00E-01	0	no
ENSACAG: 000535	CLN5	2.04E-02		integral to membrane	ENSACAT: 030710	5.00E-01	1	no

References

1 Bushmanova, E., Antipov, D., Lapidus, A., Suvorov, V. & Prjibelski, A. D. rnaQUAST: a quality assessment tool for de novo transcriptome assemblies. *Bioinformatics* **32**, 2210–2212, doi:10.1093/bioinformatics/btw218 (2016).

2 Yang, W., Qi, Y. & Fu, J. Exploring the genetic basis of adaptation to high elevations in reptiles: a comparative transcriptome analysis of two toad-headed agamas (genus *Phrynocephalus*). *PLoS One* **9**, e112218, doi:10.1371/journal.pone.0112218 (2014).

3 Murrell, B. *et al.* Gene-wide identification of episodic selection. *Mol Biol Evol* **32**, 1365–1371, doi:10.1093/molbev/msv035 (2015).

4 Murrell, B. *et al.* FUBAR: a fast, unconstrained bayesian approximation for inferring selection. *Mol Biol Evol* **30**, 1196–1205, doi:10.1093/molbev/mst030 (2013).

5 Smith, M. D. *et al.* Less is more: an adaptive branch-site random effects model for efficient detection of episodic diversifying selection. *Mol Biol Evol* **32**, 1342–1353, doi:10.1093/molbev/msv022 (2015).

6 Kosakovsky Pond, S. L. *et al.* A random effects branch-site model for detecting episodic diversifying selection. *Mol Biol Evol* **28**, 3033–3043, doi:10.1093/molbev/msr125 (2011).

7 Sun, Y. B. *et al.* Species groups distributed across elevational gradients reveal convergent and continuous genetic adaptation to high elevations. *Proceedings of the National Academy of Sciences U S A* **115**, E10634-E10641, doi:10.1073/pnas.1813593115 (2018).
